# Utilization of Mid-Thigh Magnetic Resonance Imaging to Predict Lean Body Mass and Knee Extensor Strength in Obese Adults

**DOI:** 10.3389/fresc.2022.808538

**Published:** 2022-03-24

**Authors:** Stephan G. Bodkin, Andrew C. Smith, Bryan C. Bergman, Donglai Huo, Kenneth A. Weber, Simona Zarini, Darcy Kahn, Amanda Garfield, Emily Macias, Michael O. Harris-Love

**Affiliations:** 1 Department of Physical Medicine and Rehabilitation, University of Colorado Anschutz Medical Campus, Aurora, CO, United States; 2 Division of Geriatrics, University of Colorado Anschutz Medical Campus, Aurora, CO, United States; 3 Division of Endocrinology, Diabetes, and Metabolism, University of Colorado Anschutz Medical Campus, Aurora, CO, United States; 4 Department of Radiology, University of Colorado Anschutz Medical Campus, Aurora, CO, United States; 5 Department of Anesthesia, Stanford University, Stanford, CA, United States

**Keywords:** obesity, MRI, quadriceps, intramuscular adipose tissue, convolutional neural network (CNN)

## Abstract

**Purpose:**

To train and test a machine learning model to automatically measure mid-thigh muscle cross-sectional area (CSA) to provide rapid estimation of appendicular lean mass (ALM) and predict knee extensor torque of obese adults.

**Methods:**

Obese adults [body mass index (BMI) = 30–40 kg/m^2^, age = 30–50 years] were enrolled for this study. Participants received full-body dual-energy X-ray absorptiometry (DXA), mid-thigh MRI, and completed knee extensor and flexor torque assessments *via* isokinetic dynamometer. Manual segmentation of mid-thigh CSA was completed for all MRI scans. A convolutional neural network (CNN) was created based on the manual segmentation to develop automated quantification of mid-thigh CSA. Relationships were established between the automated CNN values to the manual CSA segmentation, ALM *via* DXA, knee extensor, and flexor torque.

**Results:**

A total of 47 obese patients were enrolled in this study. Agreement between the CNN-automated measures and manual segmentation of mid-thigh CSA was high (>0.90). Automated measures of mid-thigh CSA were strongly related to the leg lean mass (*r* = 0.86, *p* < 0.001) and ALM (*r* = 0.87, *p* < 0.001). Additionally, mid-thigh CSA was strongly related to knee extensor strength (*r* = 0.76, *p* < 0.001) and moderately related to knee flexor strength (*r* = 0.48, *p* = 0.002).

**Conclusion:**

CNN-measured mid-thigh CSA was accurate compared to the manual segmented values from the mid-thigh. These values were strongly predictive of clinical measures of ALM and knee extensor torque. Mid-thigh MRI may be utilized to accurately estimate clinical measures of lean mass and function in obese adults.

## INTRODUCTION

Nearly half of Americans suffer from obesity, a condition tied to comorbidities that can lead to preventable, premature death (1, 2). Excessive adipose tissue can decrease insulin sensitivity with intramuscular adipose tissue (IMAT) demonstrating negative relationships with glucose infusion rates (3). Skeletal muscle, being the primary regulator of insulin, decreases in mass and strength within individuals with high measures of A1C, or glycated hemoglobin (4). It has been shown that obesity, or the presence of excess adipose tissue, can act as a synergist with muscular atrophy, leading to poor health-related outcomes (5, 6). As such, accessible measures to monitor muscle health should be sought for obese individuals.

Obesity is diagnosed using an individual’s body mass index (BMI), which is a metric that fails to account for high-muscle composition vs. high-fat composition. A more informative anthropometric measure is lean body mass (LBM) or appendicular lean mass (ALM), which characterizes the amount of fat-free tissue using dual-energy X-ray absorptiometry (DXA) imaging. Measures of LBM can be utilized as a predictor of chemotherapy toxicity among patients with colon cancer (7), mortality indicator among patients with hemodialysis (8), and identifier of obese individuals at risk for cardiovascular disease (9). The vast number of patient populations who benefit from the objective quantification of LBM stresses the importance of clinical access to this measure.

The growth of medical imaging has presented with overutilization of the prescription and referral of imaging, resulting in higher medical costs for patients, additional exposure to radiation, and longer time to obtain clinical outcomes of interest (10, 11). Proposed methods to reduce this are to utilize prior imaging that patients may have already received (10). It is common for patients to have existing imaging for diagnostic or screening purposes within clinical care. For example, patients often have MRI performed for cancer screening or orthopedic procedures of the lower extremities and low back (12). With existing imaging available, it provides the opportunity to obtain measures of tissue quality without supplementary referrals for DXA imaging, which induces additional exposure to radiation. Not only is MRI reliable and does not use ionizing radiation, but it may also provide accurate estimates of LBM and muscle cross-sectional area (CSA) and volume in comparison to measures obtained through DXA imaging (13, 14).

However, the manual measurement of MRI scans can be subjective and time-consuming, limiting the clinical applicability to quantify muscle CSA in order to estimate ALM. The ability to automate procedures to estimate ALM from existing medical imaging would provide a convenient option for clinicians to provide data-driven individualized care. Additionally, assessing the predictive ability of CSA to muscle strength may provide functional insights from existing imaging. Recently, convolutional neural networks (CNNs) have been reported as a viable machine learning-based approach to rapidly and accurately measure MRI of skeletal muscle (15). An available CNN to provide swift quantification of CSA to predict ALM and knee extensor toque, a proxy measure of functional status, of obese individuals would facilitate the desired data-driven individualized care of this patient population. Therefore, the purpose of the proposed research is to train and test a machine learning model to automatically measure mid-thigh muscle CSA to both provide rapid estimation of ALM and predict knee extensor torque of obese adults.

## METHODS

This was a retrospective cohort study in obese adults. All data were collected in a controlled laboratory setting and clinical imaging unit. The dependent variables for the study were manually measured mid-thigh CSA *via* MRI, ALM assessed *via* DXA imaging, and peak knee extensor and flexor torque. Independent variables were CNN values of CSA. This study was approved by the university’s institutional review board. Study abbreviations can be found in Table 1.

### Participants

All participants were recruited from a university hospital setting and the surrounding community. Participants were included if they met the following criteria from a larger parent study: BMI = 30–40 kg/m^2^, age = 30–50 years, planned physical activity <2 h/week, fasting triglyceride <400 ml/dl, and FSH < 20 IU/L. Participants were excluded if they changed oral contraceptive use during the study, were taking medications that affect glucose and lipid metabolism, had a history of thyroid disease, were pregnant, or were actively smoking.

### Knee Extensor Torque

Isokinetic, concentric knee extension and flexion were measured using an isokinetic dynamometer (Cybex NORM; Computer Sports Medicine Inc, Stoughton, MA, USA) at a speed of 60°/s from 110° of knee flexion to 0° (terminal extension). The participants completed practice trials for practice and familiarization. The participants provided maximal effort through their full range of motion for four repetitions. Measures of peak torque for the knee extension were exported from the multimode dynamometer for the right limb to demonstrate the maximum voluntary strength capacity of the knee extensors and flexors.

### Dual-Energy X-Ray Absorptiometry

Estimates of ALM were obtained *via* the whole-body DXA imaging (GE Medical Systems Ultrasound & Primary Care Diagnostics, LLC, Madison, WI, USA). A single trained DXA technician administered all DXA examinations. All data were obtained *via* GE Encore v15 SP2 software package. The body composition data collected during the DXA examinations included estimates of absolute and percentage of right leg fat mass (%), right leg lean mass (%), ALM (kg/m^2^), and total body fat (%). The ALM values were calculated as the sum of LBM in the arms and legs and scaled to height.

### Magnetic Resonance Imaging

All participants received right thigh muscle MRI scans at both study visits using a 3.0-T scanner with body coil (Siemens Syngo, Munich, Germany). The protocol details were: axial 2D gradient recalled, slice thickness = 5 mm, spacing between slices = 10 mm, field of view (FOV) = 192 × 126 mm, echo time (TE) = 1.15 ms, repetition time (TR) = 352 ms, flip angle = 9°, 24 slices, and bandwidth = 930 Hz/pixel.

Two individuals (rater 1: S.B. and rater 2: A.S.) manually selected three axial slices of the proximal thigh by identifying the MR slice just distal to the gluteus maximus tendon insertion for the right limb, using 3D Slicer (Brigham and Women’s Hospital, Harvard Medical School, Boston, MA, USA). The thigh musculature of the selected slices was then manually segmented, excluding subcutaneous adipose tissue and the femur.

### Convolutional Neural Network

A 2D U-Net CNN was created based on the manual segmentation, implemented with Python (Version 3.8, Python Software Foundation, Beaverton, OR, USA) on a computer running Ubuntu (Version16.04) and loaded with Keras (Version 2.3.1) deep learning library, with CUDA 9.1 for GPU acceleration (16). All images were zeropadded to 192 by 192 pixels to fit the input size of UNet model. The data were randomly split with 70% of the data for training, 10% for validation, and 20% for testing. Data partitions were disjoint at patient level (same patient data only exist in one of the training, validation, or testing datasets). Real-time data augmentation was performed by applying the following random image transformations: image rotation (−10° to 10°), image translation (19 pixels each direction), and image zooming (0–20%) for each epoch. Training was performed with 100 epochs, with a learning rate of 0.001. Binary cross-entropy was selected for the loss function (17). Measures of CSA (CSA_CNN), mid-thigh intramuscular adipose tissue (IMAT_CNN), and mid-thigh lean muscle (LM_CNN) were calculated [(IMAT_CNN) + (LM_CNN) = (CSA_CNN)].

### Statistical Analysis

The assumption of normality was assessed with the Shapiro–Wilk test. Levene’s test was used to assess homogeneity of the data.

Sørensen–Dice (DICE) coefficients and intersection over union (Jaccard indices, IOU) were calculated to test for agreements between the CSA_CNN, CSA_Manual: rater 1, and CSA_Manual: rater 2. DICE and IOU coefficients are two commonly reported ways to assess performance of a CNN model (18). Pearson’s *r* correlations were performed to assess the relationship between the CNN_CSA to Right_Leg_FM, Right_Leg_LM, total body fat, and ALM. Linear regression analysis was performed with ALM as the dependent variable, CNN_CSA as the independent variable, and age as a covariate.

Pearson’s *r* correlations were performed to assess the relationship between knee extensor and flexor peak torque (Nm) and the CNN_CSA. Linear regression analysis was performed with knee extensor peak torque as the dependent variable, CNN_CSA as the independent variable, and age as a covariate. To explore the influence of patient sex, the study cohort was stratified and separate regressions were performed for men and women. An *a priori* alpha was set ≤0.05 for all analyses. All statistical analyses were conducted through SPSS (Version 26; IBM Inc., Chicago, IL).

## RESULTS

A total 47 participants were enrolled and included for analyses. Patient demographics can be found in Table 2.

Agreement between the CSA_CNN, CSA_Manual: rater 1, and CSA_Manual: rater 2 were all high (DICE and IOU values all >0.90, Table 3). Pearson’s *r* correlations between CNN_CSA and DXA measures of body composition can be found in Table 4. Controlling for age, the CNN_CSA was able to predict 73% of the variance of ALM (*r*^2^ = 0.731, *p* < 0.001). For every 1 cm^2^ increase in mid-thigh CSA, ALM increased by 2% (B = 0.02, *p* < 0.001).

Pearson’s *r* correlations between CNN_CSA and knee extensor torque can be found in Table 5. Controlling for age, the CNN_CSA was able to predict 76% of the variance of knee extensor torque (*r*^2^ = 0.764, *p* < 0.001). Once stratified, this did not differ between men (*r*^2^ = 0.79, *p* < 0.001) and women (*r*^2^ = 0.72, *p* < 0.001). For every 1 cm^2^ increase in mid-thigh CSA, knee extensor peak torque increased by 4.3% (B = 0.043, *p* < 0.001).

## DISCUSSION

Individualized healthcare and treatment prescriptions are largely dependent on the access and availability to objective measurements of health. The current study was performed to develop methodology to accurately quantify LBM from existing MRI data and use this information to predict knee extensor torque. LBM estimated through our CNN model was highly accurate compared to both manual segmentation of thigh musculature, DXA measures of LBM, and DXA-measured thigh lean mass. Additionally, the CNN-quantified LBM was strongly related to knee extensor torque, a proxy measure for physical function. These results demonstrate the clinical utility of utilizing existing medical images to obtain objective measures to inform clinical decision-making.

Strong agreement was found between manually segmented measures and CNN results of mid-thigh CSA. Prior literature has shown MRI to accurately estimate LBM (14). However, methods to extract muscular CSA to estimate LBM are both time-consuming and may lack reliability between clinicians. Though homogeneity is still needed within deep learning methods (19), advances in deep learning from healthcare data present opportunity to improve the quantification of imaging outcomes. The U-net CNN model used in this study is one of the most common architectures used for segmentation tasks and has been employed in several other areas of musculoskeletal research, including the shoulder, lumbar spine, knee, and pelvis (20–23). The application of CNNs to image segmentation tasks has been a major step forward in the musculoskeletal image analysis, allowing for the automatic extraction of multiple quantitative measures of the musculoskeletal system (24, 25). The translation of these methods to the clinic has not yet been realized; several barriers to their clinical implementation remain to be overcome. Most research studies have trained and tested the models on datasets from a single site with identical imaging parameters in a homogenous participant sample. More diverse datasets are needed for both training and testing to develop analysis pipelines that generalize across patients (i.e., age, sex, and body habitus), conditions, and images (i.e., resolution, orientation, and contrast). Next, normative databases will also need to be established to interpret the clinical importance of these measures and provide clinicians with clinical cut-offs to guide musculoskeletal care. Finally, regulatory agencies will likely need to approve safety and effectiveness of these pipelines prior to their clinical implementation.

Additionally, CNN measures of LBM were found to strongly relate to DXA measures of total body fat percentage, lower limb lean mass, and ALM (Table 4). Stronger CNN relationships were present with measures of lean mass derived from DXA imaging (Right_Leg_LM and ALM, Table 4). This data may suggest more accurate estimation of lean muscle tissue compared to fat mass with machine learning methods. Prior literature (13, 14) has discovered more accurate estimates of LM and IMAT from MRI, with DXA imaging underestimating abdominal fat mass and overestimating extremity muscle mass, with this bias increasing in patients with a greater BMI (26). Underestimation of fat mass from DXA imaging may contribute to the weak relationship (*r* = 0.34, *p* = 0.04) observed in the current study. However, both total body fat and LBM are associated with an increased risk of mortality (27). The ability to predict these measures from the existing mid-thigh medical images may advance quantification practices to reduce the need for further imaging referrals. These measures may additionally be utilized to provide useful health risk information to manage patient care.

Increase in adiposity has been shown to be harmful for muscular function in addition to a greater risk of muscle degeneration throughout aging. The current study found CNN measures of LBM were highly predictive of knee extensor torque. Quadriceps strength has been shown to be greater in obese and morbidly obese individuals compared to lean individuals (28). However, when quadriceps strength is normalized by body mass, we see this comparison inverse (29). Measures of BMI, VO_2 Max_, and knee extensor torque explain 73% of the variance in the 6-min walk test within obese individuals, demonstrating the functional importance of this measure (28). Furthermore, obese individuals are at a greater risk for osteoarthritis and age-related declines in muscle function (i.e., sarcopenic obesity) (30, 31). The knee extensor torque has been found to independently increase the risk of lower extremity limitation (difficulty walking) and activities of daily living in women with osteoarthritis (32). Additionally, quadriceps strength is the best measure of age-related muscle decline and is associated with physical disability in activities of daily living (33). With the projected rise of both the obese and aging populations (34, 35), the accessibility to informative measures is vital to improve health-related outcomes.

## LIMITATIONS

Estimated values of CSA and LBM were from CNN models utilizing mid-thigh MRI of 47 obese participants. The MRI dataset was from the same scanner using the same imaging parameters; therefore, the CNN performance may not generalize to other datasets with varying imaging parameters and non-obese participants. Second, we used CSA at the mid-thigh to measure muscle size with MRI. While CSA was associated with the DXA body composition measures (total body fat percentage, lower limb lean mass, and ALM) and knee extension and flexor strength, CSA provides only an estimate of muscle size, and muscle volume measures may provide a more accurate representation of the muscle. Additionally, the flexor, extensor, and adductor compartments were segmented together and included in the CSA measure. Segmentation of the flexor, extensor, and adductor muscle groups separately could provide a more granular assessment of muscle size and composition and a stronger association with knee flexor and extension torques and is an area for future research. However, the primary goal of this project was to develop methodology access LBM measures from clinical MRI of the thigh. Knee extension and flexor strength were the only measures of functional function in the current study. Though related to outcomes of walking speed in obese patients (28), future studies should investigate more robust measures of physical function. In utilizing existing patient imaging, it is important for the clinician to consider the time since that image was taken, as the patient’s health history or composition may have changed. Prior to clinical implementation of other populations, the current study’s methodology should be assessed on a larger sample with varying imaging parameters, age, and body habitus to determine generalizability of the CNN model to the diversity seen in the clinical population.

## CONCLUSION

The CNN-measured CSA values were accurate compared to the manual segmented values from the mid-thigh. These values were strongly predictive of clinical measures of LBM, total body adiposity, and knee extensor torque. The mid-thigh imaging can be utilized to accurately estimate clinical measures of ALM and knee extensor torque in obese adults.

## Figures and Tables

**TABLE 1 |:** Abbreviations and variable descriptions from study imaging methods.

Measure	Abbreviation	Description

**Magnetic resonance imaging (MRI)**		
Cross-Sectional Area	CSA	2-Dimensional area measure of mid-thigh tissue excluding sub-cutaneous fat and the femur
Manual	CSA_Manual	Cross-sectional area measured by human tracing of mid-thigh tissue
Convolutional neural networks	CSA_CNN	Cross-sectional area measured by machine learning methods
Intramuscular adipose tissue	IMAT_CNN	Intramuscular adipose tissue measured by machine learning methods
Lean muscle	LM_CNN	Lean muscle measured by machine learning methods
**Dual energy X-ray absorptiometry (DXA)**		
Appendicular lean mass	ALM	Sum of lean body mass in the arms and legs and scaled to height
Lean body mass	LBM	Lean mass present within the entire body
Right leg fat mass	Right_Leg_FM	Fat mass present in the right leg
Right leg lean mass	Right_Leg_LM	Lean mass present in the right leg
Total body fat (%)	-	Fat mass present within the entire body expressed as a percentage of total body mass

**TABLE 2 |:** Patient demographics.

	Mean ± SD

Total patients, *n*	47
Sex (M:F)	28:19
Age (years)	39.2 ± 5.4
Height (m)	1.71 ± 0.09
Mass (kg)	104.3 ± 15.0
BMI (kg/m^2^)	35.5 ± 4.3

**TABLE 3 |:** Agreement statistics for mid-thigh cross-sectional segmentation.

	DICE	IOU

CSA_CNN vs. CSA_Manual: Rater 1	0.9649 ± 0.0135 (95%CI: 0.9362, 0.9796)	0.9324 ± 0.0249 (95%CI: 0.8801, 0.9601)
CSA_CNN vs. CSA_Manual: Rater 2	0.9558 ± 0.0242 (95%CI: 0.8930, 0.9718)	0.9163 ± 0.0403 (95%CI: 0.8067, 0.9452)
CSA_Manual: Rater 1 vs. CSA_Manual: Rater 2	0.9553 ± 0.0202 (95%CI: 0.9341, 0.9706)	0.9151 ± 0.0327 (95%CI: 0.8764, 0.9428)

DICE, Sørensen–Dice agreement coefficients; IOU, Intersection-Over-Unions.

**TABLE 4 |:** Relationships between CNN model estimates and DXA-quantified measures of body composition.

		Right_Leg_FM	Right_Leg_LM	Total Body Fat	ALM (kg/m^2^)

CSA_CNN	*r*	−0.11	**0.86**	**−0.67**	**0.87**
	*p*	0.49	**<0.001**	**<0.001**	**<0.001**
IMAT_CNN	*r*	**0.32**	−0.24	**0.57**	**−0.300**
	*p*	**0.04**	0.11	**<0.001**	**0.04**
LM_CNN	*r*	−0.14	**0.86**	**−0.71**	**0.87**
	*p*	0.38	**<0.001**	**0.04**	**<0.001**

R, right; LBM, lean body mass; CNN, convolutional neural network; CSA, cross-sectional area; IMAT, intramuscular adipose tissue. Significant values bolded (p < 0.05).

**TABLE 5 |:** Relationships between CNN model estimates and knee extensor and flexor torque.

		Raw (Nm)		Normalized (Nm/kg)	
		Knee extensor torque	Knee flexor torque	Knee extensor torque	Knee flexor torque

CSA_CNN	*r*	**0.76**	**0.48**	**0.60**	0.30
	*p*	**<0.001**	**0.01**	**<0.001**	0.07
IMAT_CNN	*r*	**−0.41**	−0.162	**−0.49**	−0.15
	*p*	**0.01**	0.33	**0.01**	0.36
LM_CNN	*r*	**0.77**	**0.48**	**0.63**	0.31
	*p*	**<0.001**	**0.01**	**<0.001**	0.06

LBM, lean body mass; CNN, convolutional neural network; CSA, cross-sectional area; IMAT, intramuscular adipose tissue. Significant values bolded (p < 0.05).

## Data Availability

The original contributions presented in the study are included in the article/supplementary material, further inquiries can be directed to the corresponding author/s.
